# Comparison of ELISpot and FluoroSpot in the Analysis of Swine Flu-Specific IgG and IgA Secretion by *in Vivo* Activated Human B Cells

**DOI:** 10.3390/cells1020027

**Published:** 2012-04-20

**Authors:** Gun Kesa, Per H. Larsson, Niklas Ahlborg, Bernt Axelsson

**Affiliations:** Mabtech AB, Box 1233, SE-13128, Nacka Strand, Sweden; E-Mails: per@mabtech.com (P.H.L.); niklas@mabtech.com (N.A.); bernt@mabtech.com (B.A.)

**Keywords:** Influenza A (H1N1), vaccination, B cells, IgG and IgA secretion, FluoroSpot, ELISpot

## Abstract

We have evaluated a novel B-cell FluoroSpot assay for the analysis of antibody responses in healthy individuals vaccinated intramuscularly with Influenza A (H1N1) antigen (Pandemrix^®^, GlaxoSmithKline). Using the FluoroSpot assay and an ELISpot assay run in parallel for comparison, we measured the frequency of cells secreting antigen-specific as well as total IgG or IgA antibodies seven days post vaccination. The assays were based on high affinity monoclonal antibodies for capture and detection of human IgG and IgA. Whereas conventional ELISpot analyzes IgG- and IgA-secreting B cells separately, fluorescent detection enabled simultaneous enumeration of B cells secreting IgG or IgA in the same well. The FluoroSpot protocol was also simpler as the assay could be performed without the need for an amplifying detection step. While having all the advantages of a conventional ELISpot assay, including high sensitivity, robustness and ease of performance, the FluoroSpot assay adds further value in reducing costs, time and material.

## 1. Introduction

There are many assays designed to measure antibody reactivity and specificity (e.g., ELISA, immunoblot, *etc.*) but only a few focuses directly on the antibody-secreting cells (ASC). One such assay is the B-cell ELISpot [1,2]. With this highly sensitive method one can, at the cellular level, identify and enumerate both the total number of ASC in a sample and those secreting antibodies to a given antigen (Ag). If B cells have been potently activated *in vivo* and have developed into ASC, they can be added to an ELISpot assay plate and incubated in cell culture medium without any additional stimulation. However, studies have shown that the time frame in which one can detect Ag-specific ASC in the peripheral blood after e.g., vaccination is narrow [3]. Typically, cells need to be collected 5 to 10 days after the administration of Ag. After this, a sharp drop in the number of circulating ASC occurs. Normally, B cells will then remain in low numbers in the circulation in the form of memory cells which require several days of stimulation *in vitro* to become ASC.

Since it was first described in 1983, the B-cell ELISpot has been performed in essentially the same way with some recent exceptions [4]. Recently, however, FluoroSpot has emerged as an attractive alternative to ELISpot [5]. In the FluoroSpot assay, secreting cells are identified using detection reagents labeled with fluorophores instead of enzymes. The fluorophore-based detection enables analysis of multiple analytes in one and the same well and hence FluoroSpot has been used to analyze dual cytokine secretion by polyfunctional T cells as well as the cytokine profile of monocyte subsets [6,7,8,9]. With regard to B cells, the use of FluoroSpot has so far been limited to a single color analysis in a study addressing the amount of antibodies secreted per B cell [10].

We have in this study evaluated a newly developed B-cell FluoroSpot assay for the analysis of human IgG- and IgA-secreting B cells activated in response to Influenza A (H1N1) vaccine (Pandemrix^®^). We measured Ag-specific as well as total IgG- and IgA-secreting B cells in peripheral blood mononuclear cells (PBMC) prepared from venous blood of six healthy individuals.

We found that the FluoroSpot technique was as sensitive as the B-cell ELISpot run in parallel and offered the additional possibility to define and enumerate, in a single well, B cells secreting antibodies of both IgG and IgA isotype. This makes the technique particularly suitable in situations where the source of cells or the amount of Ag is limited. Relevant application areas for the methods include detection of B-cell responses in various diseases and those elicited by vaccination.

## 2. Results and Discussion

### 2.1. The ELISpot and the FluoroSpot are Equally Sensitive

To evaluate the sensitivity of the new FluoroSpot assay, we measured the frequency of B cells secreting H1N1-specific IgG and IgA antibodies before and after vaccination with Pandemrix^®^ in six donors, using ELISpot in parallel. Measurement of total IgG- and IgA-ASC was performed simultaneously.

**Figure 1:**
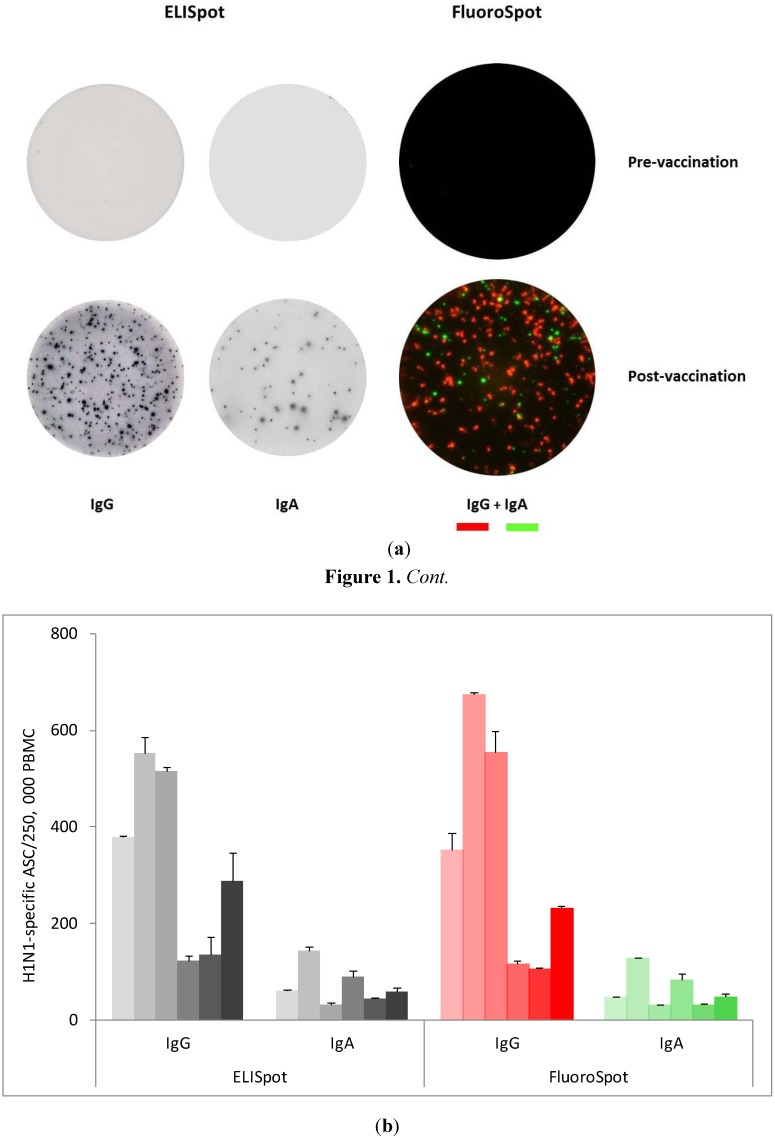
(**a**) Analysis of Influenza A (H1N1)-specific IgG- and IgA-secreting cells in ELISpot and in FluoroSpot. PBMC were collected before and seven days after vaccination with the influenza vaccine Pandemrix^®^. Ag-specific IgG- and IgA-ASC were detected using 0.75 μg/well of coated Ag. After overnight culturing of PBMC (250,000 cells/well), detection mAbs specific for IgG and IgA, and labeled with different fluorophores, were mixed together and added to the wells (FluoroSpot). In ELISpot, ASC were detected by adding biotin-labeled anti-IgG or anti-IgA detection mAbs followed by SA-ALP and a precipitating substrate. The figure shows representative examples of the vaccine-induced responses in donor 1; (**b**) Analysis of Influenza A (H1N1)-specific IgG- and IgA-secreting B cells in ELISpot and in FluoroSpot in six donors (ASC/ 250,000 PBMC). No Ag-specific ASC were seen before vaccination. Grey bars represent ELISpot whereas colored bars represent FluoroSpot. The diagram shows mean values with SD (n = 3). Each set of bars corresponds to data from donor 1–6 in numerical order from left to right.

After an overnight culture of PBMC (250,000 cells/well) in Ag-coated plates, spots representing Ag-specific ASC were, in the FluoroSpot assay, detected by adding a mixture of detection monoclonal antibodies (mAb) labeled with red fluorophore (IgG) and green fluorophore (IgA). In ELISpot, detection was made with biotinylated versions of the same antibodies with streptavidin-alkaline phosphatase (SA-ALP) added as a secondary step. An example of images showing Ag-specific FluoroSpot and ELISpot is shown in Figure 1a and the overall result for all six donors is shown in Figure 1b. As can be seen, all donors responded to Pandemrix^®^ and although the strength of the responses varied in the different donors, the frequencies of ASC were similar in the two assays. While there were no detectable spots before vaccination, the frequencies of H1N1-specific IgA-secreting B cells varied between approximately 1 in 2,000 PBMC and 1 in 8,000 in the six donors. The frequency of H1N1-specific IgG-secreting B cells was higher in all donors and ranged from 1 in 400 to 1 in 2,000 PBMC.

**Figure 2:**
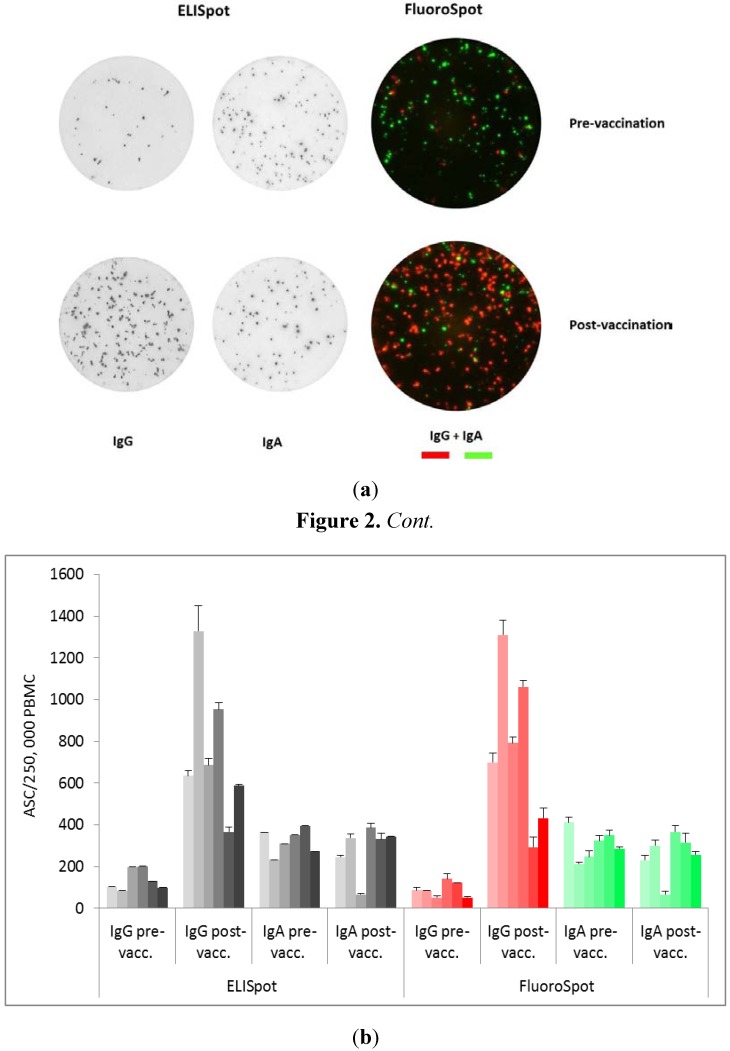
(**a**) Analysis of the total number of B cells secreting IgG and IgA after vaccination with Pandemrix^®^. The wells were coated with mAbs specific for IgG and/or IgA. After an overnight culture of PBMC (100,000 cells/well) the detection procedure was identical to that used for detection of Ag-specific ASC in Figure 1a. The images are representative examples from donor 1. (**b**) ELISpot and FluoroSpot analysis of the frequency of B cells secreting IgG and IgA before and after vaccination with Pandemrix^®^ (ASC/100,000 PBMC). The diagram shows mean values with SD (n = 3). Each set of bars corresponds to data from donor 1–6 in numerical order from left to right.

The total number of IgG- and IgA-secreting cells were also determined with the two assays and, as exemplified in Figure 2a and summarized for all six donors in Figure 2b, also here were the results very similar in the two assays.

Similar frequencies were thus recorded for FluoroSpot and ELISpot with regard to both H1N1-specific (Figure 1) and total IgG/IgA ASC (Figure 2), demonst rating that the sensitivity of the two assays was comparable. In the FluoroSpot, there was no difference in the number or intensity of spots in wells where the two detection reagents for IgA and IgG were added together *versus* when they were used separately (data not shown).

When comparing the ELISpot and the FluoroSpot assays, it should be noted that the ELISpot was based on a two-step detection system, *i.e.*, a biotinylated detection mAb followed by SA-ALP, whereas the FluoroSpot was done in one step with mAbs directly labeled with fluorophores. A two-step detection system was chosen for ELISpot since enzyme-conjugated mAbs generally have a lower sensitivity compared to two-step detection systems. However, despite the fact that the ELISpot protocol included an additional amplification step, the two assays displayed comparable sensitivity.

### 2.2. IgA- and IgG-ASC before Vaccination

In contrast to the lack of H1N1-specific ASC before vaccination, all donors had significant numbers of IgG- and IgA-secreting cells of unknown specificity prior to the vaccination (Figure 2a and 2b). The frequencies of pre-vaccination ASC were about 1 in 2,000 for IgG and about 1 in 800 PBMC for IgA. These ASC probably represent B cells producing so-called natural antibodies which are suggested to serve as a first line of defense against infection. Natural antibodies typically recognize common pathogen-associated epitopes and have been shown to be protective *in vivo* [11].

### 2.3. Increased Frequency of Total IgG- But Not IgA-ASC after Vaccination

In the case of IgG, vaccination also led to an increase in the number of ASC which could not be fully accounted for by the increase in H1N1-specific ASC. Thus, as seen in Figure 2b, there was a strong increase in the frequency of IgG-secreting cells after vaccination in all six donors, something that was not observed for IgA. One reason for this increase in total IgG-ASC may be a non-specific stimulatory effect of the AS03 adjuvant formulation in the vaccine (squalene, DL-α-tocopherol and polysorbate 80). However, it has also been shown that influenza as such, as well as other infections, may give rise to a polyclonal activation and antibody secretion by B cells [12].

Still, almost half of the circulating total ASC were vaccine specific with an average ratio of Ag-specific per total ASC of 0.45 for IgG and 0.4 for IgA. This is not surprising since the PBMC were analyzed without additional activation *in vitro* and thus only *in vivo*-activated ASC were detected in the assays. In healthy individuals, unless they have been recently vaccinated, active ASC are generally found at low numbers in the blood.

## 3. Experimental Section

### 3.1. Cells

PBMC were prepared from venous blood of healthy human volunteers after informed consent, before and seven days after vaccination with Pandemrix^®^ (GlaxoSmithKline, Solna, Sweden). The blood was collected in sodium citrate and PBMC separated by centrifugation on Ficoll-Paque™ Plus (GE Healthcare Life-Sciences, Uppsala, Sweden) according to the recommendation of the manufacturer.

### 3.2. Reagents

RPMI1640, Penicillin/Streptomycin, HEPES, and low endotoxin FCS were all purchased from Invitrogen Life Technologies (Carlsbad, CA, USA). Coating and detection mAbs for ELISpot (Anti-IgG, anti-IgA, biotinylated anti-IgG and –IgA; cat. no 3850-2AW and 3860-2AW, respectively) and for FluoroSpot (anti-IgG-red fluorophore and anti-IgA-green fluorophore; cat. no FS-05R06G) were all obtained from Mabtech (Nacka Strand, Sweden). SA-ALP, 5-bromo-4-chloro-3-indolyl phosphate/nitro-blue tetrazolium (BCIP/NBT) substrate and FluoroSpot enhancer were all from Mabtech.

### 3.3. ELISpot/FluoroSpot Assay

Low-fluorescent 96-well PVDF membrane plates (Millipore, Bedford, MA, USA) were pre-wetted with 20 μL of 35% ethanol/well for 1 min and thereafter washed five times with sterile water. For detection of Ag-specific B cells, Pandemrix^®^ Ag was added at 0.75 μg/well and for enumeration of total IgG/IgA-secreting cells, anti-IgG and/or anti-IgA capture mAbs were diluted in sterile PBS and added at 1.5 μg/well. After incubation overnight at 4 °C, the coated wells were washed with sterile PBS and blocked for at least 1 h with culture medium. After removal of the blocking medium, 100 μL/well of PBMC was added (100,000 cells/well for determining the total number of IgG and IgA secreting cells; 250,000 cells/well for the analysis of Ag-specific IgG- and IgA-ASC). Each sample was added in triplicates and the plates were thereafter incubated overnight at 37 °C in a humidified atmosphere with 5% CO_2_.

After incubation, the cells were removed by washing with PBS using an automated plate washer (Bio-Tek Instruments Inc., Winooski, VT, USA). Biotinylated anti-IgG or anti-IgA mAbs for ELISpot or a mixture of fluorophore-labeled anti-IgG (red) and anti-IgA mAb (green) for FluoroSpot were diluted to 1 μg/mL each in PBS containing 0.5% FCS and added to each well. After incubation for 2 h at room temperature followed by a washing step, ELISpot wells were incubated with SA-ALP for 1h at room temperature. After incubation, the plates were again washed in the plate washer with PBS followed by addition of BCIP/NBT substrate. The reaction was stopped after 10 min. by extensive washing in tap water followed by drying.

After the 2 h incubation step, the washed FluoroSpot plates were treated with 50 μL/well of Fluorescence enhancer for 15 min. at room temperature. After tapping against paper, the plastic underdrain was removed and the plate was left to dry in the airstream of a hood for 60 min.

Analysis and counting of spots were performed in an ELISpot/FluoroSpot reader system (Multispot reader Spectrum, AID, Strassberg, Germany). Fluorescent spots were analyzed using separate filters for FITC and Cy3.

## 4. Conclusions

The FluoroSpot method was as sensitive as the B-cell ELISpot run in parallel and offered the additional advantage to define and enumerate, in a single well, B cells secreting antibodies of both IgG and IgA isotype. The FluoroSpot protocol also led to a simpler and more rapid analysis by reducing the number of assay steps.

Fluorescent detection also opens up the possibility of detecting more than two isotypes or subclasses of antibodies in a single well. This makes the technique particularly suitable in situations where the source of cells and/or the amount of Ag is limited.
